# Inhibitors against Two PDZ Domains of MDA-9 Suppressed Migration of Breast Cancer Cells

**DOI:** 10.3390/ijms24043431

**Published:** 2023-02-08

**Authors:** Heng Tang, Lei Wang, Shuju Li, Xiaoli Wei, Mengqi Lv, Fumei Zhong, Yaqian Liu, Jiuyang Liu, Bangguo Fu, Qizhi Zhu, Dan Wang, Jiajia Liu, Ke Ruan, Jia Gao, Weiping Xu

**Affiliations:** 1 Institute of Intelligent Machines, Hefei Institutes of Physical Science, Chinese Academy of Sciences, Hefei 230031, China; 2 Division of Life Sciences and Medicine, University of Science and Technology of China, Hefei 230027, China; 3 Department of Pharmacology, University of Colorado Anschutz Medical Campus, Aurora, CO 80045, USA

**Keywords:** NMR fragment-based screening, MDA-9, PDZ domain inhibitor, complex structure, paramagnetic relaxation enhancement

## Abstract

Melanoma differentiation-associated gene 9 (MDA-9) is a small adaptor protein with tandem PDZ domains that promotes tumor progression and metastasis in various human cancers. However, it is difficult to develop drug-like small molecules with high affinity due to the narrow groove of the PDZ domains of MDA-9. Herein, we identified four novel hits targeting the PDZ1 and PDZ2 domains of MDA-9, namely PI1A, PI1B, PI2A, and PI2B, using a protein-observed nuclear magnetic resonance (NMR) fragment screening method. We also solved the crystal structure of the MDA-9 PDZ1 domain in complex with PI1B and characterized the binding poses of PDZ1-PI1A and PDZ2-PI2A, guided by transferred paramagnetic relaxation enhancement. The protein–ligand interaction modes were then cross-validated by the mutagenesis of the MDA-9 PDZ domains. Competitive fluorescence polarization experiments demonstrated that PI1A and PI2A blocked the binding of natural substrates to the PDZ1 and PDZ2 domains, respectively. Furthermore, these inhibitors exhibited low cellular toxicity, but suppressed the migration of MDA-MB-231 breast carcinoma cells, which recapitulated the phenotype of MDA-9 knockdown. Our work has paved the way for the development of potent inhibitors using structure-guided fragment ligation in the future.

## 1. Introduction

Human proteomes contain approximately 600,000 protein–protein interactions (PPIs) [1] to mediate cellular function. The dysfunctions of PPIs are closely related to various diseases; consequently, PPIs have gained increasing attention as an emerging frontier of drug discovery [2]. PDZ domains are small, globular PPI domains containing approximately 90 amino acids that form a classical two α-helices (α1, α2) and six β-strands (β1–β6) structure [3,4,5,6]. The PDZ proteins play essential roles in cell trafficking, channel surface retention, and neuronal signaling [7,8]. They also regulate cytoskeletal dynamics via interactions with cell junctions, cell polarity, and cell migration [9,10]. PDZ proteins are divided into three classes based on the motifs of the target peptides, i.e., class I (x-S/T-x-Φ-COOH), class II (x-Φ-x-Φ-COOH), and class III (x-D/E-x-Φ-COOH), where Φ represents a hydrophobic residue. The human PDZ domain-containing protein, melanoma differentiation-associated gene 9 (MDA-9, also known as SDCBP), recognizes class I and class II peptides [11,12]. MDA-9 contains four domains, the N-terminal (1–109aa), PDZ1 (110–193aa), PDZ2 (194–274aa), and the C-terminal (275–298aa) [13]. The PDZ2 domain of MDA-9 binds to c-Src, while the PDZ1 domain binds to TGF-β, EGFR, and IGF-1R proteins [4,6,10,14].

Through the interactions between the PDZ domains and the substrates, MDA-9 tandem PDZ domains are involved in syndecan-mediated signaling to the cytoskeleton and perform a variety of physiological and cellular functions [15,16,17], e.g., cell–cell adhesion, trans-membrane trafficking, immune regulation, autophagy, ubiquitination, neural development, and exosome control [18,19,20,21]. The dysregulation of MDA-9 drives tumorigenesis; for example, MDA-9 is overexpressed in melanoma, breast cancer, glioma, and urothelial cell carcinoma [22,23,24,25]. MDA-9 is involved in multiple signaling pathways that involve FAK, PIP2, c-Src, p38, MAPK, NF-κB, AKT, SP1, IGFBP2, SPRR1B, and EGFR [13,26,27,28,29], and has been demonstrated to drive the invasion and migration of cancer cells, e.g., small cell lung cancer [30]. Furthermore, the genetic silencing of either single or tandem PDZ domains of MDA-9 leads to an accumulation of cells in G1 phase and enhances p21 and p27 expression, which eventually suppresses cancer cell proliferation [31,32]. Clinical studies identified the correlation between MDA-9 expression and the mortality of patients with cancer [33]. Hence, these findings underpinned the pursuit of MDA-9 PDZ domains as a potential therapeutic target.

The discovery of inhibitors targeting the PDZ domain remains challenging because of the shallow and dynamic binding interface. Despite the discovery of inhibitory peptides or peptidomimetic compounds against PDZ domains, small molecules are highly desirable due to their membrane permeability and reduced risks of immune reactions or biodegradation. PDZ1i, developed by Fisher et al., was demonstrated to be able to bind to the MDA-9 PDZ1 domain with an affinity of 21 μM, but did not bind significantly to the MDA-9 PDZ2 domain. PDZ1i is a suitable potential pharmacological tool for investigating the role of the PDZ1 domain of MDA-9 in cellular mechanisms and vivo efficacy studies. For example, PDZ1i inhibited the invasion of glioma cells and improved survival rate in brain-tumor-bearing mice [6]. PDZ1i also exhibited in vivo activity against prostate cancer or neuroblastoma [18,34]. However, the lack of structural studies on the interaction mode of the inhibitor PDZ1i to the MDA-9 PDZ1 domain has further limited the development of potent drug-like compounds. Moreover, a small pharmacological inhibitor, C58, was demonstrated to weakly bind to the PDZ2 domain of MDA-9 in vitro and selectively inhibit the cancer exosomes pathway [22]. Herein, we identified four hits—PI1A, PI1B, PI2A, and PI2B—targeting the PDZ1 and PDZ2 domains of MDA-9, using NMR chemical shift perturbation (CSP) [35]. Next, we depicted the interaction patterns of the hits and MDA-9 PDZ domains using crystallography or NMR methodology. The binding modes of these inhibitors were further validated by mutagenesis. PI1A and PI2A inhibited the binding of PDZ1 and PDZ2 domains to natural substrates, respectively, and, in turn, suppressed the migration of MDA-MB-231 cells.

## 2. Results

**Figure 1:**
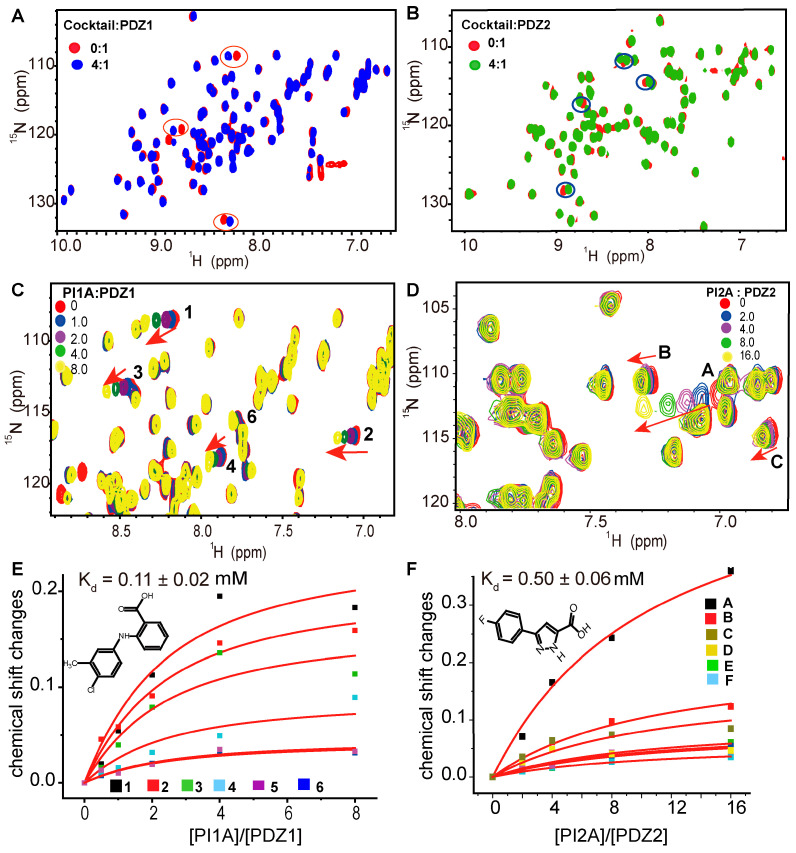
Fragment-based screening against the PDZ1 and PDZ2 domains of MDA-9. (**A**) Superimposition of the ^1^H-^15^N heteronuclear single quantum coherence (HSQC) spectra of MDA-9 PDZ1 domain upon the addition of the compound cocktail. Ligand/protein molar ratios are annotated. (**B**) Superimposed HSQC spectra of MDA-9 PDZ2 domain. (**C**) Chemical shift perturbations of MDA-9 PDZ1 domain induced by PI1A. (**D**) The ^1^H-^15^N HSQC spectra of MDA-9 PDZ2 domain upon titration of PI2A. Upon titrating PI1A and PI2A to MDA-9 PDZ1 and PDZ2 domain, respectively, some of these perturbed residues are zoomed in the boxed regions (**C**,**D**), where arrows are marked to indicate the trend of the chemical shift changes. The perturbed residues of MDA-9 PDZ1 domain were denoted by numbers and the perturbed residues of MDA-9 PDZ2 domain were denoted by letters. (**E**) The binding affinity of PI1A is determined by best fitting of the dose-dependent CSPs of MDA-9 PDZ1 domain. (**F**) Determination of the binding affinity of PI2A to MDA-9 PDZ2 domain by CSPs. The red solid line represents the dose-dependent titration curve for each disturbed residue of MDA-9 PDZ1 and PDZ2 domains. The numbers 1–6 represent all perturbed residues of MDA-9 PDZ1 domain, while the letters A–F represent all perturbed residues of MDA-9 PDZ2 domain.

The protein-observed CSPs were deployed to screen a 540 low-molecular-weight drug library and an 890-fragment library against the MDA-9 PDZ1 and PDZ2 domains, respectively. To enhance the screening throughout, cocktails with 10 compounds each were added to the ^15^N-labeled MDA-9 PDZ1 or PDZ2 domain at a ligand/protein molar ratio of 4:1, respectively (Figure 1A,B). A cocktail that induced significant CSPs of two or more residues of the MDA-9 PDZ1 or PDZ2 domain was singled out for further deconvolution. Each component of such a cocktail was titrated to the ^15^N-labeled MDA-9 PDZ1 or PDZ2 domain, respectively (Figure 1C,D). PI1A and PI1B bound to the MDA-9 PDZ1 domain with an affinity of 0.11 and 0.34 mM, respectively, determined from the dose-dependent CSPs (Figure 1E and Figure S1A,B). The similar CSP patterns indicated that PI1A and PI1B bound to the same pocket of the MDA-9 PDZ1 domain. Meanwhile, another two hits, PI2A and PI2B, were identified for the PDZ2 domain of MDA-9, which possessed a common pharmacophore of pyrazole carboxylic acid and perturbed a similar set of residues. The binding affinities of PI2A and PI2B to MDA-9 PDZ2 are 0.50 mM and 0.61 mM, respectively, which were determined by an NMR titration assay. (Figure 1F and Figure S1C,D). Consistent with the low sequence identity of 26% between the MDA-9 PDZ1 and PDZ2 domains (Figure S2), PI1A did not bind to the MDA-9 PDZ2 domain (Figure S3A), while PI2A bound to the MDA-9 PDZ1 domain at a weak affinity over 1 mM (Figure S3B).

**Figure 2:**
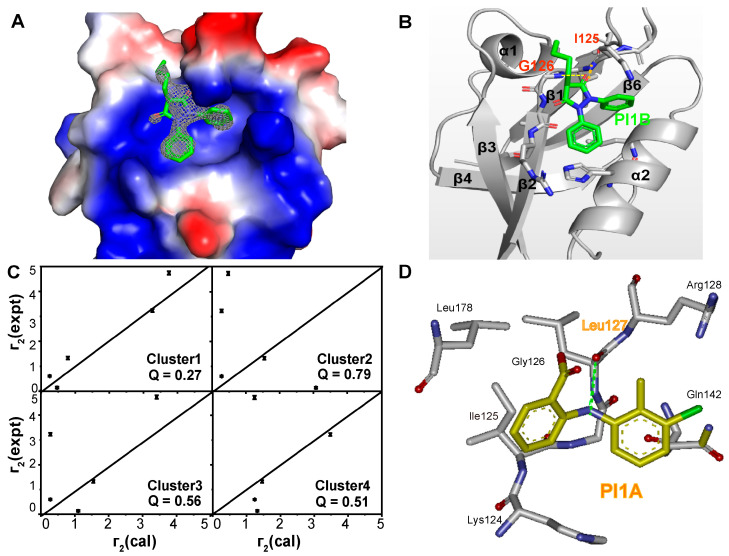
The binding modes of PI1B and PI1A to MDA-9 PDZ1 domain. (**A**) The electrostatic surface representation of the crystal structure of MDA-9 PDZ1 domain in complex with PI1B (PDB ID: 8HCK). The 2Fo-Fc electron-density map of PI1B is contoured at 1σ (gray mesh). (**B**) Detail interactions between protein and ligand. The secondary structure elements are labeled in the crystal structure of MDA-9 PDZ1. The green dash lines represent hydrogen bonds. (**C**) Correlations between the experimental (*Γ*_2(expt)_) and back-calculated (*Γ*_2(cal)_) PRE values for the lowest energy pose in each cluster. The black solid squares indicate the experimental and back-calculated PRE values for each H atom of PI1A and annotated with experimental errors. (**D**) Detailed interactions between MDA-9 PDZ1 domain (carbon atoms in gray) and PI1A (yellow).

To gain insight into the binding details, we solved the crystal structure of the MDA-9 PDZ1 domain in complex with PI1B (Figure 2A, Table S1). The complex structure indicated that PI1B was bound in a typical substrate recognition groove between the α2 helix and β2 strand of the MDA-9 PDZ1 domain (Figure 2A,B). This groove was mainly composed of the residues K124, I125, G126, L127, R128, Q142, H175, and L178. PI1B formed two hydrogen bonds with the backbone of Ile125 and Gly126 (Figure 2B), and residue G126 was also a conserved residue for binding to the substrate peptide. Notably, the side chains of residues Q142, R128, and K124 were rearranged upon ligand binding, relative to the free form.

The binding model of the MDA-9 PDZ1 domain in complex with PI1A was generated by molecular docking and guided by transferred paramagnetic relaxation enhancement (PRE) restraints. Transferred PRE describes the difference of transverse relaxation rates of the ligand in the presence of the diamagnetically or paramagnetically labeled protein, which was proportional to the inverse sixth power of the distance between the paramagnetic center and the ligand atom of interest [36]. Since the perturbed residues in PI1A were similar to those in PI1B, the size of the AutoDock grid map was reduced to encompass these perturbed residues. The conformations of PI1A docked with MDA-9 PDZ1 were classified into four clusters based on docking energy. We then introduced the C166S mutagenesis, such that the remaining C118 residue was chemically linked to methanethiosulfonate (MTSL). Both the HSQC and CD spectra of the C118/C166S mutant or MTSL-modified proteins showed that the overall folding of these proteins was not disturbed, compared to wild-type proteins. However, a significant signal decay of the residues spatially adjacent to C118 residues could be observed in the HSQC spectrum of the MTSL-labeled protein (Figures S4A and S5). The titration of PI1A against the MTSL-labeled C118/C166S mutant perturbed a similar set of signals, with a binding affinity of 0.25 mM (Figure S4B). The transverse relaxation rate (R2) was assessed based on the exponential signal decay of the transverse relaxation delay for each proton of PI1A, for which each proton chemical shift was assigned (Figure S4C,D). The paramagnetic R2 values of PI1A were measured in the presence of the MTSL-labeled MDA-9 PDZ1 domain (Figure S4C and Table S2). In contrast, the diamagnetic R2 values were detected after the addition of sufficient amounts of vitamin C to reduce the MTSL sample to a diamagnetic state (Figure S4D and Table S2). The Q-factor was used to assess the agreement between experimental and back-calculated PRE values (Figure S6), i.e., a lower Q-value indicated a higher agreement and a better-fitting binding conformation. Cluster 1 presented the best-fitting one, with a Q value significantly lower than the others in all four clusters (Figure 2C). This model indicated that PI1A formed a direct hydrogen bond with the residue L127 of the MDA-9 PDZ1 domain (Figure 2D).

**Figure 3:**
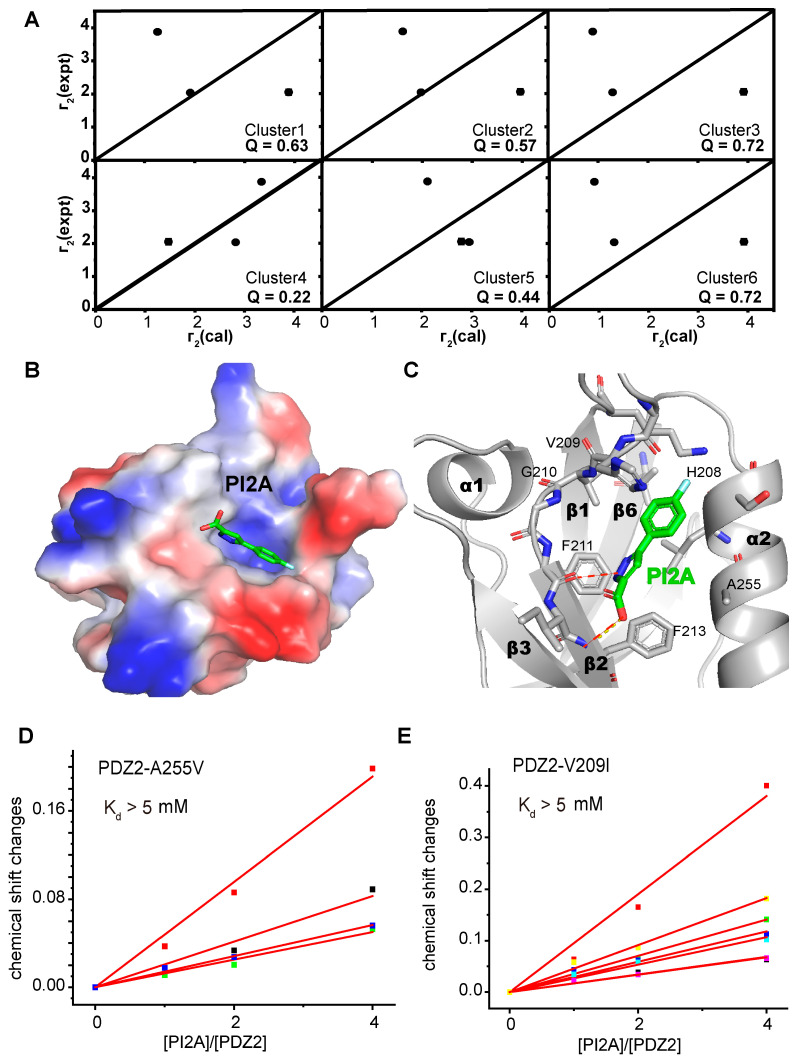
Binding modes of PI2A for MDA-9 PDZ2 domain. (**A**) Correlations between the experimental and back-calculated PRE values for the lowest energy pose within each cluster. The Q values for each pose are annotated. The black solid squares indicate the experimental and back-calculated PRE values for each H atom of PI2A and annotated with experimental errors. (**B**) The lowest energy binding pose of the PI2A and MDA-9 PDZ2 domain in cluster 4. The electrostatic surface of MDA-9 PDZ2 domain is represented. PI2A is in a green stick representation. (**C**) Detailed interactions between PI2A and conserved residues within 5Å of MDA-9 PDZ2 domain. (**D**,**E**) Binding affinities of PI2A to the A255V or V209I mutant of MDA-9 PDZ2 domain can be best-fitted from the dose-dependent titration curves (red solid lines) for the disturbed residues simultaneously. Fitting multiple titration curves simultaneously provides a more accurate estimate of *K_d_*. The different colored squares in (**D**,**E**) represent the chemical shift changes for each residue at different concentrations. Chemical shift changes were calculated as [(Δδ_H_)^2^ + (Δδ_N_/5)^2^]^1/2^, where Δδ_H_ and Δδ_N_ denote the chemical shift changes for ^1^H and ^15^N, respectively.

Similarly, we also attempted to elucidate the interaction mode of the MDA-9 PDZ2 domain and the fragment screening hits using crystallographic or NMR spectroscopy methods. Unfortunately, neither PI2A nor PI2B crystallized with the MDA-9 PDZ2 domain, probably due to their limited binding affinity and solubility. Autodock 4.2 (developed by Olson Laboratory of Scripps Research Institute, La Jolla, CA, USA) was used to model the structure of PI2A or PI2B in complex with the unbound MDA9 PDZ2 domain. Meanwhile, we referred to more distance information between the protein and the ligand to further filter out the best-fit poses. However, since the ^1^H spectrum of PI2B had only one proton (Figure S7) in the aromatic region (6–10 ppm), it did not provide sufficient NMR constraints to reduce the possible docking poses, which limited the use of NMR methodology to further obtain a model of PI2B in complex with the MDA-9 PDZ2 domain. In contrast, PI2A had more ^1^H signal at 6–10 ppm and provided more structural information. As such, we used molecular docking, guided by NMR restraints, to describe the binding mode of PI2A and the MDA-9 PDZ2 domain. The crystal structure of the MDA-9 PDZ2 domain in complex with its peptide substrate (PDB ID: 1OBY) revealed three key interactions with conserved residues, V209, G210, and F211, of MDA-9 [37]. The V209A and G210A mutants exhibited reduced binding affinities to PI2A (Figure S8A,B), indicating that PI2A bound to the same pocket as the one for the substrate recognition. With this defined binding site, the Autodock algorithm was applied to generate six clusters with the lowest docking energy. The C239S/D256C mutant of the MDA-9 PDZ2 domain was then MTSL labeled, which did not disrupt the overall folding of the PDZ2 domain (Figure S9A) and the inhibitor binding affinity (Figure S9B). The T2 relaxation spectra of PI2A were acquired upon the titration of the MTSL-tagged PDZ2 domain (Figure S9C,D). The transferred PRE values of PI2A were measured in the presence of paramagnetically or diamagnetically labeled MDA-9 PDZ2 domains (Table S3). Cluster 4 represented the best-fitting pose with the lowest Q value among all six clusters (Figure 3A and Figure S10). This conformer indicated that PI2A formed two hydrogen bonds with the backbone of residues F211 and F213, and a sigma-π interaction with residue H208 (Figure 3B,C). This binding model was further validated, as the H208A mutant of the MDA-9 PDZ2 domain was incapable of binding to PI2A (Figure S11). Interestingly, the A255V and V209I mutants of the MDA-9 PDZ2 domain, observed in patients with gastric or head and neck cancers (data from TCGA, PanCancer Atlas), also exhibited a reduced affinity (Kd > 5 mM) (Figure 3D,E), probably due to the induced steric repulsions.

**Figure 4:**
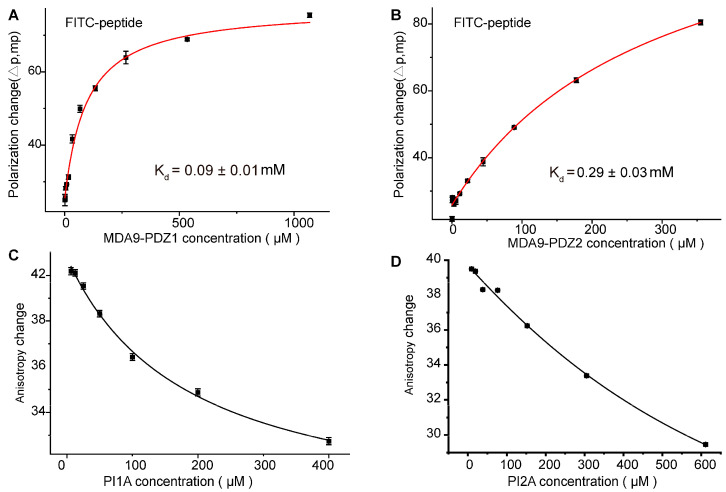
PI1A/PI2A blocking the binding between MDA-9 PDZ1/PDZ2 domain and their peptide substrate. (**A**,**B**) The binding affinities of MDA-9 PDZ1 or PDZ2 domain to the FITC-labeled peptide (TNEFYF) determined by fluorescence polarization experiments, respectively. Error bars represent the standard deviations estimated from three biological duplicates. (**C**) Displacement of FITC-labeled peptide from MDA-9 PDZ1 domain upon titration of PI1A. (**D**) Displacement of FITC-labeled peptide from MDA-9 PDZ2 domain upon titration of PI2A. The assay was performed by adding 50 μL PI1A or PI2A at the indicated concentrations, respectively, to 150 μL FITC-peptide/protein complex, incubating for 2 h, and measuring anisotropy changes. Errors are estimated from three biological duplicates.

To assess the inhibitory activity of PI1A and PI2A, we carried out fluorescence depolarization (FP) assays to determine the binding between the MDA-9 PDZ1 or PDZ2 domain and the fluorescein isothiocyanate (FITC)-labeled TNEFYF peptide competed by PI1A or PI2A, respectively. The binding affinities of the peptide for the MDA-9 PDZ1 and PDZ2 domains were 0.094 and 0.29 mM, respectively (Figure 4A,B). The competitive FP assays further confirmed that PI1A or PI2A competed in a dose-dependent manner with the peptide substrate for binding to the grooves of the MDA-9 PDZ1 or PDZ2 domain, respectively (Figure 4C,D). It was determined that the binding affinities of PI1A to the PDZ1 and PI2A to the PDZ2 domain were 0.17 mM and 0.51 mM, respectively, according to previously reported competitive FP equations [38]. These values were in agreement with the dissociation constants (Kd) determined by the NMR titration.

**Figure 5:**
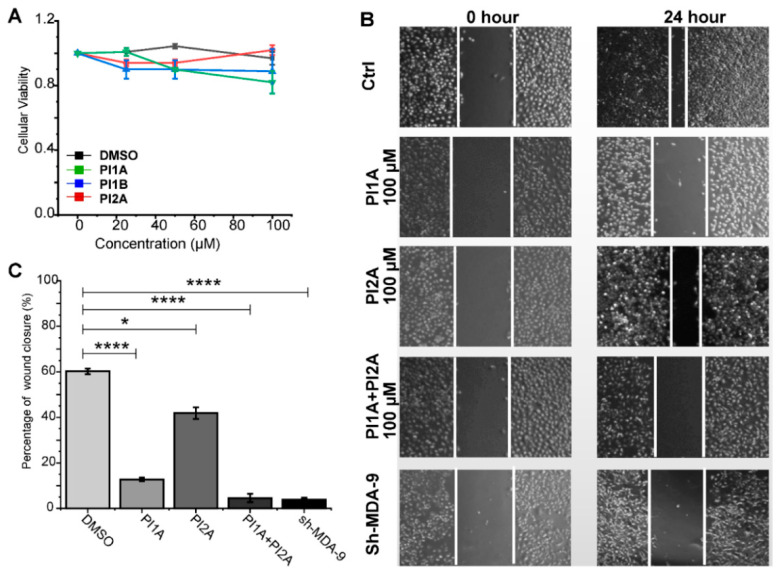
PI1A and PI2A inhibit the migration of MDA-MB-231 cells. (**A**) Viability of MDA-MB-231 cells assayed by CCK8 after treatment of PI1A, PI1B, and PI2A for 48 h. (**B**) Wide-angle micrographs of MDA-MB-231 cells treated by DMSO, PI1A (100 μM), or PI2A (100 μM) for wound healing assays. Cell migration recorded at 0 and 24 h. (**C**) Statistics of the wound closure relative to control (DMSO-treated cells). The mean ± standard deviations were estimated from three biological duplicates. The *p* values were retrieved from the one-way analysis of variance (ANOVA) with a Bonferroni post-test (* *p* < 0.05, **** *p* < 0.0001).

To assess the cellular activity of these MDA-9 inhibitors, we tested their biological effects on the proliferation and migration of the breast cancer cell MDA-MB-231. The cell counting kit-8 (CCK8) assay suggested that PI1A, PI1B, and PI2A were not toxic (Figure 5A), while the wound-healing assays demonstrated that PI1A or PI2A alone suppressed the migration of MDA-MB-231 cells (Figure 5B). Accordingly, the combination of PI1A and PI2A inhibited migration more effectively than the treatment of a single inhibitor (Figure 5C), and PI1B also demonstrated a similar synergetic effect with PI2A (Figure S12). Our data recapitulated the phenotype MDA-9 knockdown (Figure 5C and Figure S13 and Table 1). They suggested that the PDZ1 and PDZ2 domains of MDA-9 may have had a synergetic function in cellular migration, and a covalent linkage of PI2A and PI1A/PI2B could significantly improve the potency of the MDA-9 inhibitors.

## 3. Materials and Methods

### 3.1. Cloning, Expression, and Protein Purification

The PDZ1 domain (residues 113–192), PDZ2 domain (residues 197–273) and tandem PDZ structural domain (residues 113–273) of MDA-9 were cloned into the pGEX4T-1 vector (GE Healthcare, Shanghai, China) with a tobacco erosion virus (TEV) cleavage site. All mutants of the MDA-9 were generated using the Mutant BEST kit (Takara) and confirmed by DNA sequencing. Next, the construct was transformed into Escherichia coli Rosetta (DE3), cultivated in LB media, and incubated at 37 °C. Protein expression was induced at OD 600 of 1.0 using 0.2 mmol/L IPTG at 16 °C for 24 h. The bacteria were harvested by centrifugation (5000 rpm for 10 min), resuspended in lysis buffer A (25 mM Tris, 500 mM NaCl, pH 7.5), and then lysed by high-pressure crushing. We purified GST-tagged proteins from pretreated bacterial lysates using GSTrap FF (GE Healthcare, Chicago, IL, USA), then treated them with TEV to cleave the N-terminal GST tag. The protein was further purified by size exclusion chromatography using a HiLoad 16/60 Superdex 75 column (GE Healthcare, Shanghai, China). The purified protein was stored in buffer B (20 mM HEPES, 200 mM NaCl, 1 mM EDTA, 1 mM DTT, pH 7.0). For ^15^N-labeled proteins, the cells were first grown in LB media, harvested when OD600 reached 0.8 and then transferred to ^15^N-enriched SV40 medium. The cells were induced by 0.2 mM IPTG to express the target protein. The purified proteins were concentrated in a HEPES buffer (20 mM HEPES, 100 mM NaCl, 1 mM EDTA, 1 mM DTT, pH 7.0).

### 3.2. NMR Fragment-Based Screening

NMR fragment-based screening was performed at 25 °C using an Agilent 700 MHZ spectrometer equipped with a 96-well autosampler (Agilent Technologies, Santa Clara, CA, USA). ^1^H-^15^N HSQC spectra were acquired from the ^15^N-labeled MDA-9 PDZ1 and PDZ2 domains (0.05 mM). A fragment library of 890 compounds (Chembridge, SanDiego, CA, USA) and an approved drug library of 540 compounds (TargetMol, Boston, MA, USA) were pooled into cocktails, respectively, and each cocktail contained 10 compounds at 0.2 mM [39,40]. The ^1^H-^15^N HSQC spectra were measured on an Agilent 700 MHz spectrometer at ligand/protein molar ratios ranging from 0.0 to 4.0.

### 3.3. NMR Chemical Shift Perturbation

The ^15^N-labelled PDZ1 and PDZ2 domains of MDA-9 were concentrated in the HEPES buffer (20 mM HEPES, 100 mM NaCl, 1 mM EDTA, 1 mM DTT, pH 7.0) to 0.05 mmol/L. The screening fragment compounds were then titrated to these ^15^N-labeled MDA-9 PDZ1 or PDZ2 domain, respectively. Each HSQC profile was collected (at molar ratios of 0.0, 2.0, 4.0, 8.0, 16.0, and 32.0 at 25 °C) using an Agilent 700 MHz spectrometer. NMR spectra were analyzed using Sparky. The chemical shift changes (Δδ), relative to the free form of the PDZ1 or PDZ2 domain, were calculated as follows:

(1) $$\Delta \delta =\sqrt{{\left(∆\delta_{H^{1}}\right)}^{2}+{\left(0.2{\Delta \delta }_{N^{15}}\right)}^{2}}$$

where $∆\delta_{H^{1}}$ and ${\Delta \delta }_{N^{15}}$ denote the chemical shift changes of ^1^H and ^15^N, respectively.

According to this equation, the binding constants can be best fitted with a binding mode of 1:1:

$${\Delta \delta }_{\mathrm{obs}}={\Delta \delta }_{max}\frac{\left({\left[P\right]}_{t}+{\left[L\right]}_{t}+K_{d}\right)-\sqrt{{\left({\left[P\right]}_{t}+{\left[L\right]}_{t}+K_{d}\right)}^{2}-4{\left[P\right]}_{t}{\left[L\right]}_{t}}}{2{\left[P\right]}_{t}}$$

where [*P*]*_t_* and [*L*]*_t_* represent the concentrations of the protein and ligand, respectively. Δδ_obs_ represents the observed chemical shift changes, relative to the free-state protein.

### 3.4. Crystallization, Data Collection, and Structure Determination

The MDA-9 PDZ1 domain was concentrated to 15 mg/mL in a HEPES buffer (20 mM HEPES, 100 mM NaCl, 1 mM EDTA, 1 mM DTT, pH 7.0), then centrifuged at 13,000 rpm for 30 min at 4 °C. We discarded the precipitate. Crystals of the PDZ1 domain, in complex with PI1B, were grown at 4 °C using the hanging drop vapor diffusion by mixing 1 μL of mix with 1 μL of reservoir buffer (0.1 M MES monohydrate, 1.8 M Ammonium sulfate, 0.01 M Cobalt (II) chloride hexahydrate, pH 6.5) for 7–15 days. These crystals were flash frozen in liquid nitrogen using a cryoprotectant (20% glycerol added to the reservoir solution). Datasets for X-ray diffraction were collected at the Shanghai Synchrotron Radiation Facility via the BL18U beamline. HKL2000 was used to index, integrate, and scale all datasets. Phenix’s eLBOW software was used to prepare PI1B. As a search model with molecular substitution, Phaser MR4 was used to solve the composite structures of MDA-9 PDZ1 and PI1B. Coot5 was used to model the structure, followed by Refmac56 and Phenix7 for refinement. The crystal diffraction data and refinement statistics are summarized in Table S1. All structure maps were generated using PyMOL (http://www.pymol.org/ accessed on 10 October 2022).

### 3.5. Molecular Docking

AutoDock is a powerful tool for molecular docking [41]. Mutant residues were used as constraints to establish the binding pocket for AutoDock. The PDZ1 and PDZ2 domains of MDA-9 (PDB ID: 1OBY) were obtained from the PDB database, respectively, and prepared according to the following instructions. First, the PDB structures of the proteins were filled with hydrogen atoms. Then, all water and precipitates were removed. The associated missing side chains were repaired, and atomic bumps were removed using a 100-fold energy minimization algorithm. The MDA-9 PDZ1 and PDZ2 domains were imported into AutoDock as starting structures, respectively, while PDB files for PI1A or PI2A were generated by PRODRG. A cubic grid box was defined as the docking site on top of the bond slot. In accordance with RMSD threshold, the docking calculation was performed by the AutoDock server. This clustered the 100 docking structures of the PDZ1 domain into 4 clusters and the 100 docking structures of the PDZ2 domain into 6 clusters.

### 3.6. Transferred PRE

The ^15^N-labeled PDZ1 C118/C166S mutant and the PDZ2 C239S/D256C mutant were expressed by the method described above. After the dilution of the protein solution to 0.5 mg/mL with a 1-fold excess of Tris(2-carboxyethyl) phosphine (TCEP), the solution was incubated for 2 h at room temperature. The protein solution was then incubated overnight at 4 °C with an excess of 5-folds methionine spin label (MTSL). The excess MTSL molecules were removed by dialysis. In order to confirm the chemical attachment of MTSL to the mutant, ^1^H-^15^N HSQC spectra were obtained. The MTSL-labeled PDZ1 or PDZ2 domains of the mutants were used to determine that the NMR titration experiments of PI1A or PI2A did not alter the binding mode. MTSL-labeled PDZ1 or PDZ2 mutant proteins (200 μM) were titrated into a solution of PI1A or PI2A (0.2 mM), respectively. WATERGATE spectra were collected using T2 delays of 0.008 and 0.08 s. Vitamin C was then added, in excess of five times the amount of MTSL, and the reaction was incubated overnight. WATERGATE spectra with T2 delays of 0.04 and 0.8 s were recorded. The experimental error was estimated by repeating the experiment. ACD Lab was used to integrate the peaks between 6 and 8 ppm of the WATERGATE spectrum of PI1A or PI2A.

*R*_2_ was best fitted to an exponential decay equation as follows:

(2) $$I_{t}=I_{0}e^{-R_{2}t}$$

In this case, *I_t_* and *I*_0_ represent the intensities measured at the relaxation T2 delays of *t* and 0 s, respectively.

It was determined that the transferred PRE *Γ*_2_ would be as follows:

(3) $$\Gamma_{2}=R_{2\left(para\right)}-R_{2\left(dia\right)}$$

Here, *R*_2(*dia*)_ and *R*_2(*para*)_ refer to the *R*_2_ values measured in diamagnetic and paramagnetic states, respectively. The errors in *Γ*_2_ were calculated as follows:

(4) $$\sigma_{\;\left(\Gamma_{2}\right)}=\frac{1}{T_{b}-T_{a}}\sqrt{{\left(\frac{\sigma_{dia}}{I_{dia}\left(T_{a}\right)}\right)}^{2}+{\left(\frac{\sigma_{dia}}{I_{dia}\left(T_{b}\right)}\right)}^{2}+{\left(\frac{\sigma_{para}}{I_{para}\left(T_{a}\right)}\right)}^{2}+{\left(\frac{\sigma_{para}}{I_{para}\left(T_{b}\right)}\right)}^{2}}$$

*Γ*_2_ of the *i*th atom of PI1A or PI2A was correlated with the complex of PI1A-PDZ1 or PI2A-PDZ2 as follows:

(5) $$\Gamma_{2\left(i\right)}=\frac{A}{r_{ei}^{6}}$$

where *r_ei_* denotes the distance between the Cγ atom of the residue C118 (PDZ1) or the residue D256 (PDZ2) to *i*th atom of PI1A or PI2A. The constant *A* was determined by nuclear and protein properties, as well as the bound fractions of PI1A and PI2A.

The *Q* value was calculated to assess the agreement between the experimental and calculated *Γ*_2_ values:

(6) $$Q=\sqrt{\frac{\sum_{i}{\left(\Gamma_{2\left(i\right)}^{calc}-\Gamma_{2\left(i\right)}^{\exp t}\right)}^{2}}{\sum_{i}{\left(\Gamma_{2\left(i\right)}^{\exp t}\right)}^{2}}}$$

with $\Gamma_{2\left(i\right)}^{calc}$ and $\Gamma_{2\left(i\right)}^{\exp t}\;$ denoting the calculated and experimental *Γ*_2_ values for the 1st atom in PI1A or PI2A, respectively.

### 3.7. Fluorescence Polarization (FP)

The wild-type or mutant proteins of the MDA-9 PDZ1 and PDZ2 domains were prepared at various concentrations, ranging from 0 to 600 μM, as described above. The FITC-labelled peptide (sequence TNEFYFGSGS) was diluted to 500 nM in a HEPES buffer (20 mM HEPES, 200 mM NaCl, pH 7.0). The FITC-labelled peptide was mixed with different concentrations of protein and incubated for 30 min. At 25 °C, the samples were excited at a wavelength of 485 nm and detected at a wavelength of 525 nm using a CLARIOstar (BMG LABTECH, Offenburg, Germany) plate reader. The experiments were performed in triplicate. The following equation was used to fit the binding constant in a 1:1 binding mode:

(7) y=(((K+L+x)−((K+L+x)^2−4×L×x)^0.5)/(2×L))×(Ab−Af)+Af

where *y* represents the value of anisotropy, *x* represents the concentration of protein, *L* represents the concentration of the FITC-peptide, and *K_d_* represents the equilibrium dissociation constant of the FITC-peptide.

For competitive FP experiments with the FITC-peptide, the PDZ1 domain concentration was set at 80 μM and the PDZ2 domain concentration at 250 μM. After that, each aliquot of the 200 μL sample contained 500 nM of the FITC-peptide, 80 μM of the PDZ1 or 250 μM of the PDZ2 domain, as well as different concentrations of PI1A or PI2A. A concentration range of 0–200 μM was administered for PI1A while a concentration range of 0–600 μM was administered for PI2A. Samples were repeated 3 times for each experimental group. The dissociation constants for the interactions between ligands and proteins were derived from the complete competitive FP equation proposed by Gerhard Wagner et al. (Biochemistry 2004). The *K_d_* of the compounds for competitive FP was fitted using the following equation [38]:

(8) $$\begin{array}{c} y=\left(2\times {\left({\left(K1+K2+L+x-R\right)}^{\wedge }2-3\times \left(\left(x-R\right)\times K1+\left(L-R\right)\times K2+K1\times K2\right)\right)}^{\wedge }0.5\times \cos((\arccos((-2\times \right.\\ {\left(K1+K2+L+x-R\right)}^{\wedge }3+9\times \left(K1+K2+L+x-R\right)\times \left(\left(x-R\right)\times K1+\left(L-R\right)\times K2+K1\times K2\right)-27\times \\ \left(-K1\times K2\times R\right))/\left(2\times \left({\left(K1+K2+L+x-R\right)}^{\wedge }2-3\times (\left(x-R\right)\times K1+\left(L-R\right)\times K2+K1\times \right.\right.\\ \left.\left.\left.\left.\left.{K2))}^{\wedge }1.5\right)\right)\right)/3\right)-\left(K1+K2+L+x-R\right)\right)/\left(3\times K1+2\times \left({\left(K1+K2+L+x-R\right)}^{\wedge }2-3\times (\left(x-R\right)\times K1+\left(L-R\right)\right.\right.\\ \times K2+K1\times {K2))}^{\wedge }0.5\times \cos\left(\left(\arccos\left(\left(-2\times {\left(K1+K2+L+x-R\right)}^{\wedge }3+9\times \left(K1+K2+L+x-R\right)\times (\left(x-R\right)\right.\right.\right.\right.\\ \times K1+\left(L-R\right)\times K2+K1\times K2)-27\times \left(-K1\times K2\times R\right))/\left(2\times \left({\left(K1+K2+L+x-R\right)}^{\wedge }2-3\times (\left(x-R\right)\right.\right.\\ \left.\left.\left.\left.\left.\;\times K1+\left(L-R\right)\times K2+K1\times {K2))}^{\wedge }1.5\right)\right)\right)/3\right)-\left(K1+K2+L+x-R\right)\right)\times \left(Ab-Af\right)+Af \end{array}$$

where *y* represents the value of anisotropy, *x* represents the concentration of the compounds PI1A and PI2A, *L* represents the concentration of the FITC-peptide, *R* represents the concentration of protein, *K*1 denotes the equilibrium dissociation constant of the FITC-peptide, and *K*2 represents the dissociation constant and indicates the probability that the small molecule to be fitted will compete for binding to the protein with the FITC-peptide.

### 3.8. Cell Viability Assay

The MDA-MB-231 cells were obtained by a gift. CCK8 assay was used to assess the effects of PI1A or PI2A on the viability of MDA-MB-231 cells. Cells were placed in 96-well plates and treated with different concentrations of DMSO, PI1A, or PI2A for 48 h. Then, 10 μL of CCK8 solution was added to the wells. The 96-well plates were incubated in a CO_2_ incubator at 37 °C for another 1 h. OD values were measured at 450 nm using a CLARIOstar (BMG LABTECH) plate reader.

### 3.9. Wound-Healing Assay

MDA-MB-231 cells were spread in 6-well plate culture dishes, where sh-MDA-9 cells were used as a positive control. DMSO (control), PI1A 100 μM, PI2A 100 μM, and both PI1A and PI2A 100 μM were added to the MDA-MB-231 cells, respectively. On newly grown monolayer cells, 10 L of the tip was used to line the central area of cell growth. Next, excess cells were washed away with PBS buffer. The cells were then cultured for the experimentally-determined period of time (0 h or 24 h). Lastly, the cell culture plates were removed separately and the peripheral cells were observed under a microscope to determine whether they had migrated to the scratched area.

### 3.10. Quantitative Real-Time PCR Analysis

MDA-9 shRNA and control shRNA were purchased from Open Biosystems. HEK293T cells were seeded in a 6-well dish and then transfected with 1μg of retroviral DNA encoding MDA-9 vector or control vector, 1 μg packaging plasmid mix (0.8 μg psRAX2 and 0.2 μg pMD2.G) to generate lentiviruses. Virus infection was performed in MDA-MB-231 cells for 48 h. Add puromycin to screen for virus-infected cells. Cells were then collected and total RNA was extracted. RNA extraction was performed using SV Total RNA (Promega, Beijing, China). cDNA was reserved from total RNA by using the First Strand cDNA Synthesis Kit (Thermo, Shanghai, China). The Real-time PCR (Applied Biosystems ABI 7500, Waltham, MA, USA) and SYBR-Green PCR Master Mix (Takara, Beijing, China) were used for amplification and detection. GAPDH was used as the normalization control of the amplifications.

## 4. Conclusions

MDA-9 is an adaptive scaffolding protein that exerts its diverse functions by interacting with downstream proteins. MDA-9 plays a crucial regulatory role in various tumorigenic pathways, making it an emerging therapeutic target. Although PDZ1i has been shown to be an effective pharmacological tool to study the biological function of the MDA-9 PDZ1 domain in cells and in vivo, structural studies of its interaction with the MDA-9 PDZ1 domain are absent. In addition, C58 is a weak inhibitor that only targets the MDA-9 PDZ2 domain. The highlight of our study was the screening of inhibitors against the PDZ1 and PDZ2 domains of MDA-9, respectively. Meanwhile, we investigated the interaction pattern of the inhibitors with the proteins using biophysical approaches. The details of these interactions will provide a fine-grained structural basis for the subsequent development of the bivalent state inhibitors of the MDA-9 PDZ tandem. The fragment-screened hit PI1A and PI2A were shown to inhibit the binding of the MDA-9 PDZ domain to natural substrates through competitive FP assays, respectively. Furthermore, both compounds, PI1A and PI2A, demonstrated low toxicity to the MDA-MB-231 cells and impaired cell migration, consistent with the results of MDA-9 knockdown. Overall, we identified two compounds that bound to the natural substrate pocket of the tandem PDZ domains of MDA-9. Using structure-guided fragment linking, we envision the development of potent bivalent state inhibitors that will provide a powerful strategy for cancer therapy in the future.

## Figures and Tables

**Table 1:** Primers used on this report.

Amplicon	Forward Primer	Reverse Primer
MDA-9	TTGAACATATTATTAAGCGGATG	TGAACAAGGTATCAGATGAAAGAG
GAPDH	TGTTGCCATCAATGACCCCTT	CTCCACGACGTACTCAGCG

## Data Availability

Structure data are deposited in the Protein Data Bank with the accession code 8HCK.

## Supplementary Materials

The following supporting information can be downloaded at: https://www.mdpi.com/article/10.3390/ijms24043431/s1.

## Abbreviations

MDA-9	Melanoma differentiation-associated gene 9
FBLD	Fragment-based lead discovery
CSPs	Chemical shift perturbations
HSQC	Heteronuclear single-quantum coherence
NMR	Nuclear Magnetic Resonance
PRE	Paramagnetic relaxation enhancement
FP	Fluorescence depolarization
FITC	Fluorescein isothiocyanate
CCK8	Cell counting kit-8
sh-MDA-9	MDA-9 knockdown
