# Supplementary material for: Inhibitors against Two PDZ Domains of MDA-9 Suppressed Migration of Breast Cancer Cells
Source: Int J Mol Sci. 2023 Feb 8;24(4):3431. doi: 10.3390/ijms24043431 (PMC9964117; doi:10.3390/ijms24043431)
Supplement: Supplementary file 1 [file ijms-24-03431-s001.zip › ijms-2157284 - supplementary.pdf]

Supporting Information

Inhibitors against Two PDZ Domains of MDA-9 Suppressed Migration of Breast Cancer Cells

Heng Tang[1,2], Lei Wang[2], Shuju Li[2], Xiaoli Wei[2], Mengqi Lv[2], Fumei Zhong[2], Yaqian Liu[2], Jiuyang Liu[3], Bangguo Fu[2], Qizhi Zhu[1,2], Dan Wang[2], Jiajia Liu[2], Ke Ruan[2], Jia Gao[2*] and Weiping Xu[1,2*]

[1] Institute of Intelligent Machines, Hefei Institutes of Physical Science, Chinese Academy of Sciences, Hefei 230031, China

[2] Division of Life Sciences and Medicine, University of Science and Technology of China, Hefei 230027, China

[3] Department of Pharmacology, University of Colorado Anschutz Medical Campus, Aurora, CO 80045, USA

* Correspondence: jiagao@ustc.edu.cn (J.G.); weipingx@ustc.edu.cn (W.X.)

Supporting Tables

Table S1. X-ray crystallography data collection and refinement statistics of MDA-9 PDZ1 domain in complex with PI1B.

**PDB ID**	8HCK
**Data collection**	
Space group	$P4_122$
Cell dimensions	
a, b, c (Å)	49.353, 49.353, 75.254
α, β, γ (°)	90, 90, 90
Wavelength (Å)	0.97915
Resolution* (Å)	25.59 - 2.0
	(2.072 - 2.0)
Completeness (%)	100 (100)
Redundancy	24.6 (25.2)
R_{sym} or R_{merge} (%)	5.29 (30.59)
$I/\sigma I$	45.41 (12.15)
CC1/2	1.000 (0.991)
**Refinement**	
No. of reflections used/free	6731/935
R_{work}/R_{free}	23.56/26.45
R.m.s. deviations	
Bond lengths (Å)	0.008
Bond angles (°)	0.879
B-factors (Å^2)	
Protein	33.09
Ligand	59.70
Water	34.14
No. of atoms	
Protein	615
Ligand	45
Water	19
Ramachandran plot	
Favored/allowed/outlier (%)	97.5/2.5/0.0

* Values in parentheses are for highest-resolution shells.

Table S2. Paramagnetic relaxation enhancement of PI1A transferred from spin-labeled MDA-9 PDZ1 domain.

**C118**	**H6**	**H8**	**H9**	**H7/14**	**H15/16**
**$R_{2(para)}$**	1.78	1.27	4.98	6.78	3.29
**$R_{2(dia)}$**	0.83	0.67	0.66	0.96	0.88
**$\Gamma 2$**	0.95	0.60	4.32	5.82	2.41
**σ**	0.04	0.03	0.06	0.07	0.06

Table S3. Paramagnetic relaxation enhancement of PI2A transferred from spin-labeled MDA-9 PDZ2 domain.

**D256C**	**H4**	**H7/11**	**H8/10**
**$R_{2(para)}$**	2.74	4.73	2.56
**$R_{2(dia)}$**	0.69	0.85	0.52
**$\Gamma 2$**	2.05	3.88	2.04
**σ**	0.07	0.04	0.03

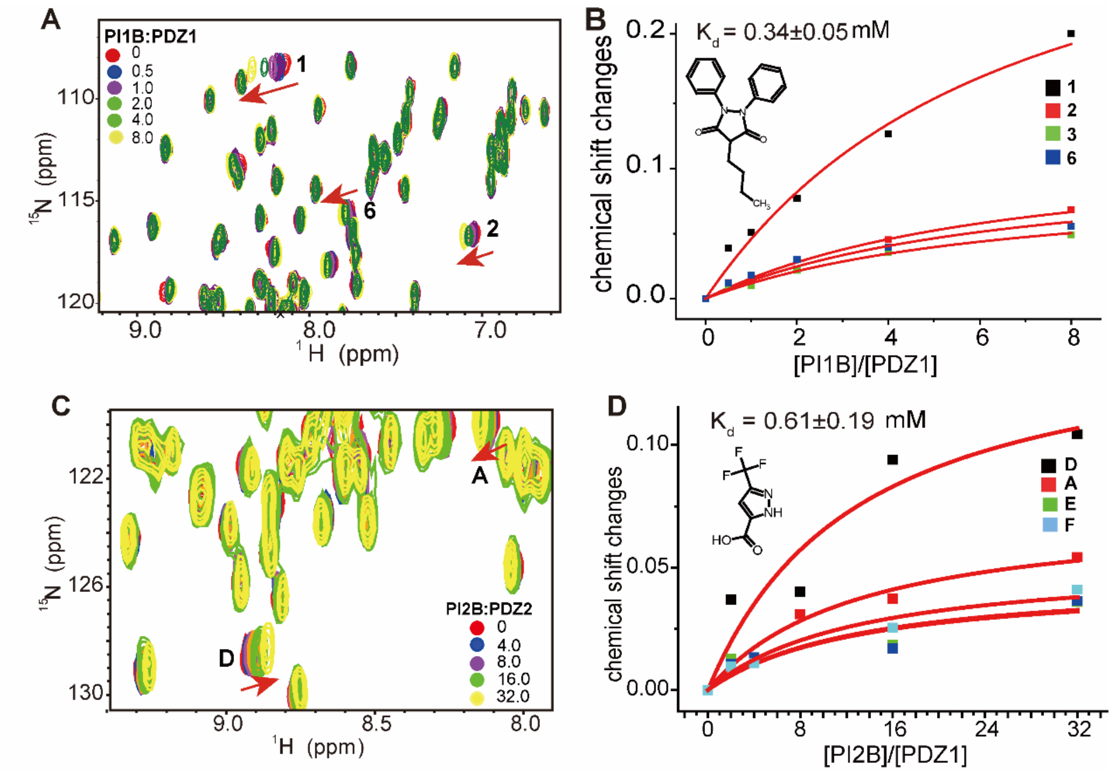

Supplementary Figure S1. The ^{1}H-^{15}N HSQC spectra of MDA-9 PDZ1 or PDZ2 domain upon titration of PI1B (A) or PI2B (C), respectively, at the annotated ligand/protein molar ratios. A number of disturbed residues are zoomed in A and C, where arrows are marked to show the trend of chemical shift changes. The binding affinity of PI1B (B) or PI2B (D) to MDA-9 PDZ1 or PDZ2 domain, respectively, was determined by the dose-dependent CSPs.

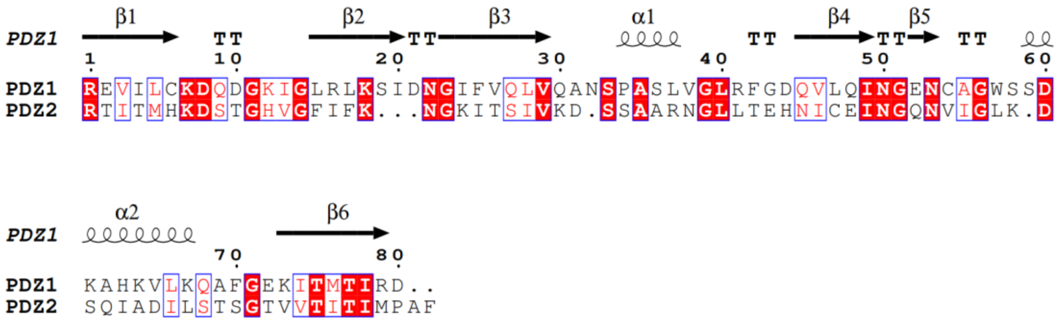

Supplementary Figure S2. Sequence alignment of the PDZ1 and PDZ2 domains of MDA-9.

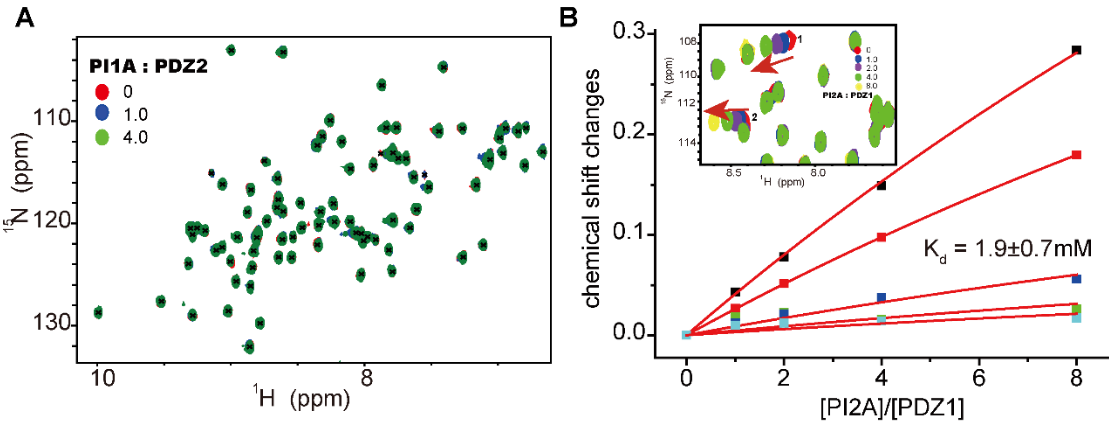

Supplementary Figure S3. Binding affinity of PI1A (A) to MDA-9 PDZ2 domain and PI2A (B) to PDZ1 domain at annotated ligand/protein molar ratios.

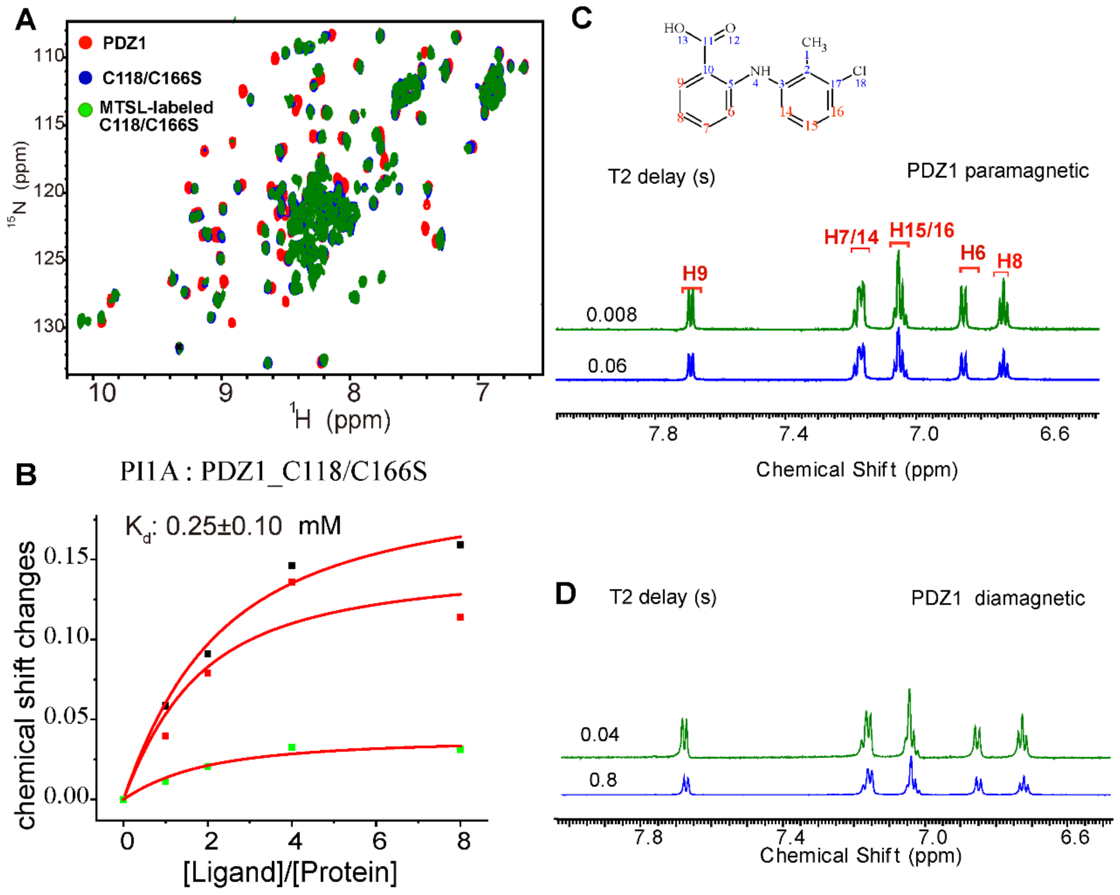

Supplementary Figure S4. Determination of paramagnetic relaxation enhancement of PI1A transferred from MTSL-labeled MDA-9 PDZ1 domain. (A) Superimposition of ^{1}H-^{15}N HSQC spectra of the wild-type, C118/C166S mutant, and the MTSL-labeled C118/C166S mutant of MDA-9 PDZ1. (B) Binding affinity between PI1A and MTSL-labeled C118/C166S mutant of MDA-9 PDZ1 domain determined by dose-dependent CSP. (C) The 1H chemical shift assignments of PI1A. Proton T2 spectrum of PI1A (0.1 mM) in the presence of MTSL-labeled MDA-9 PDZ1 domain (C) and after Vitamin C reduction (D). Annotated T2 delays.

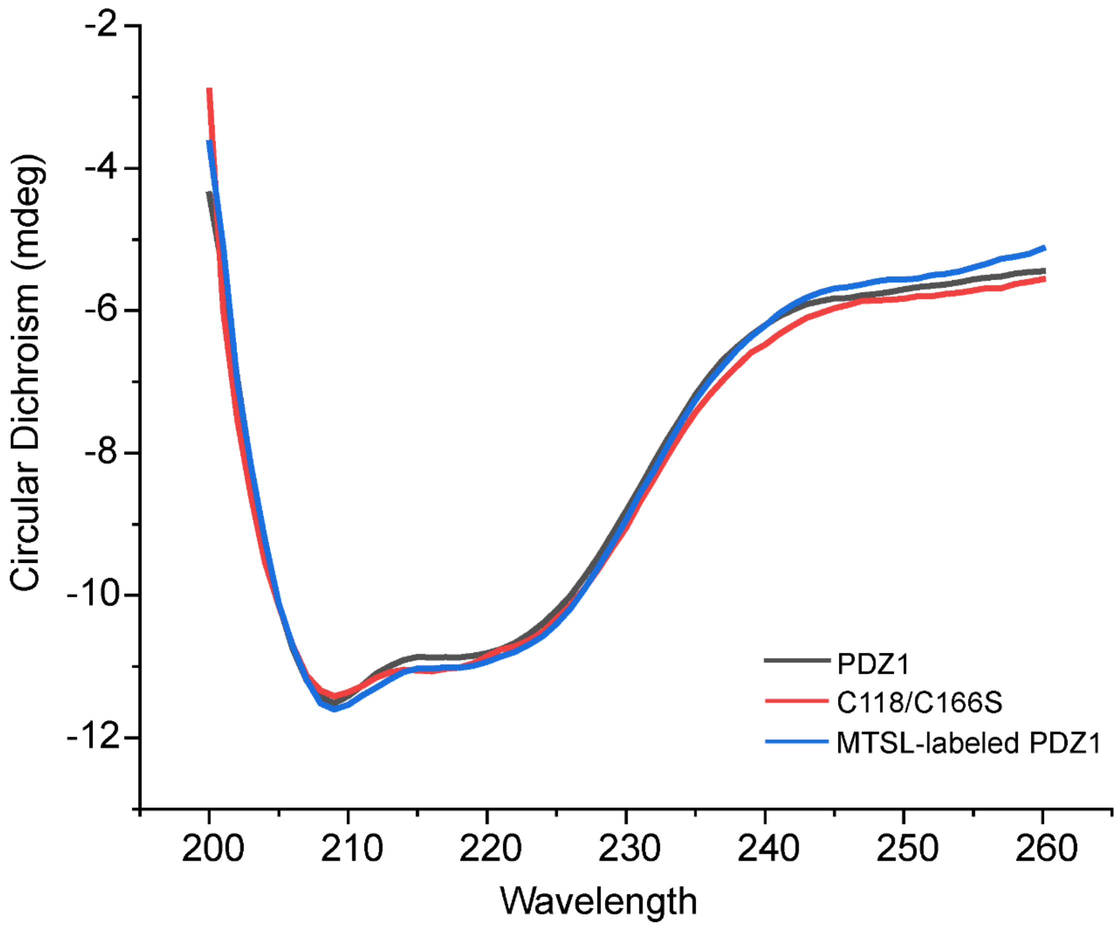

Supplementary Figure S5. Circular dichroism spectra of the three proteins include wild-type PDZ1 (black curve), C118/C166S mutant (red curve), and MTSL-labeled C118/C166S mutant of MDA-9 PDZ1.

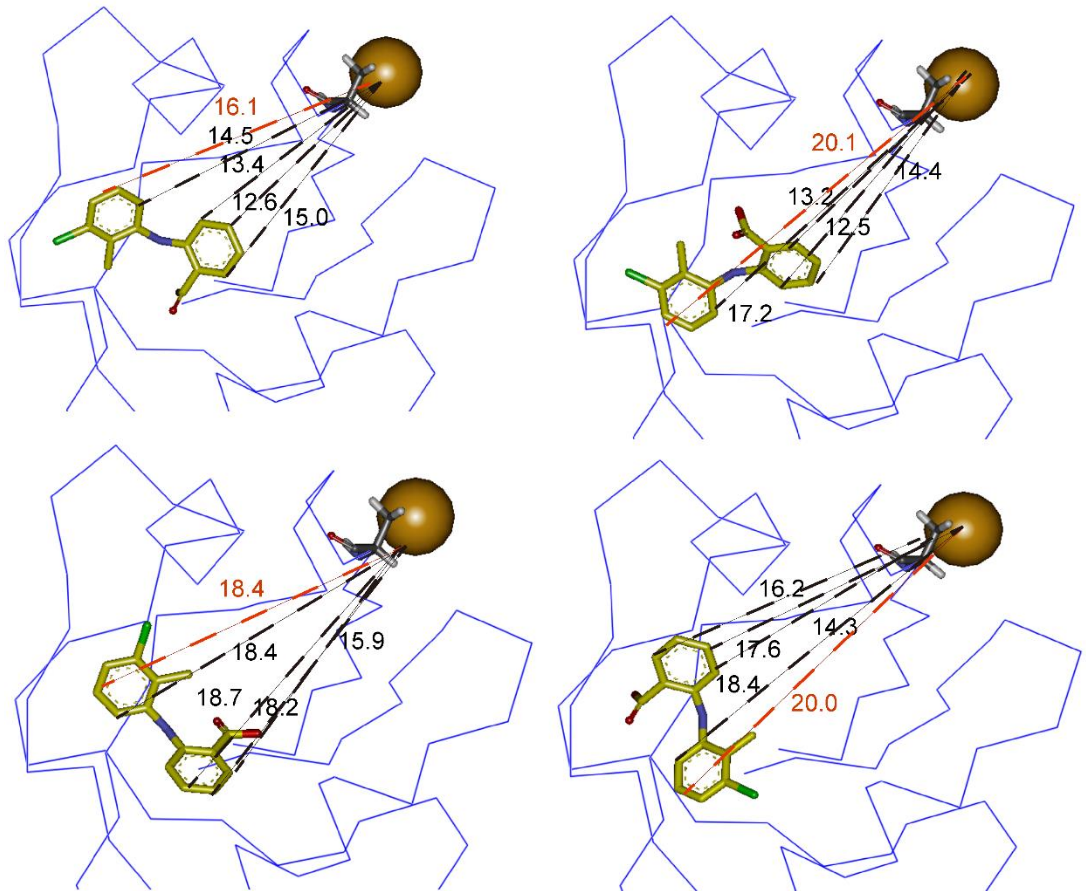

Supplementary Figure S6. The distance between the hydrogen atoms on the aromatic ring of PI1A and the paramagnetic center was measured in each docking cluster. The position of the sulfur atom (orange sphere) of residue C118 (gray stick) of MDA-9 PDZ1 domain represents approximately the position of the nitroxide radical in the paramagnetic center. The black dash lines represent the distances with values annotated. The orange dashed lines represent the linear average of the distances from the H15 and H16 atoms to the S atom of C118, respectively.

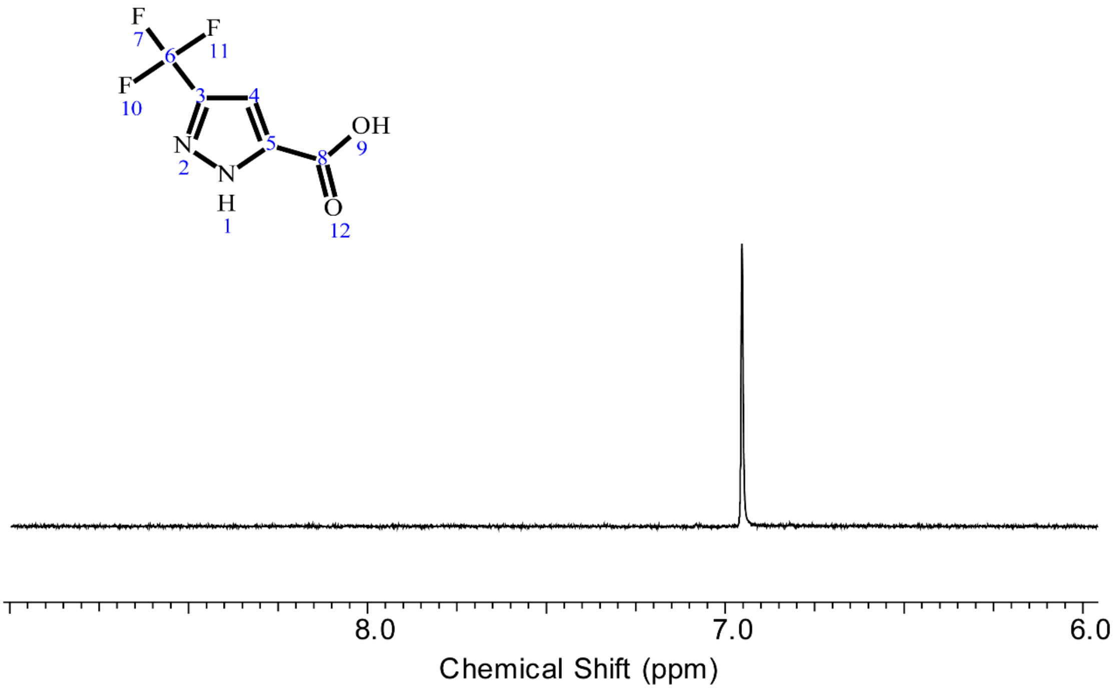

Supplementary Figure S7. The ^{1}H chemical shift of PI2B.

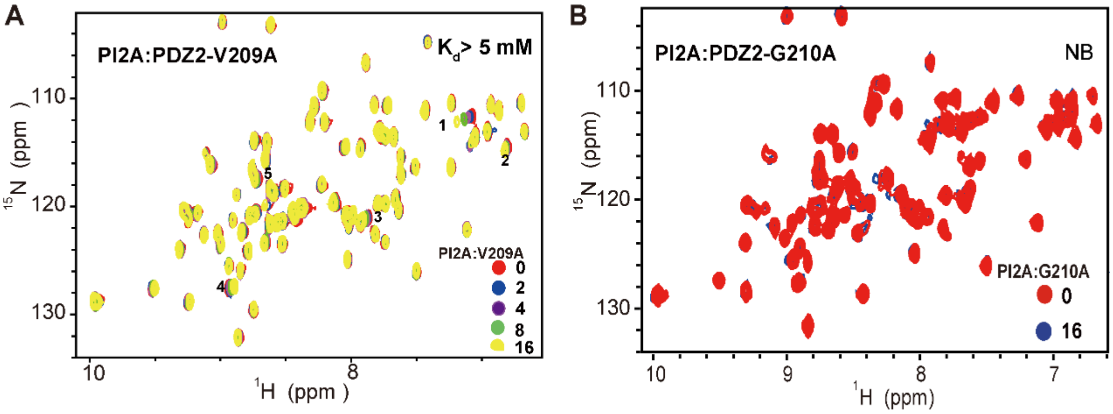

Supplementary Figure S8. Binding assays of PI2A to the V209 and G210A mutants of MDA-9 PDZ2 domain, respectively. Ligand/protein molar ratios are annotated.

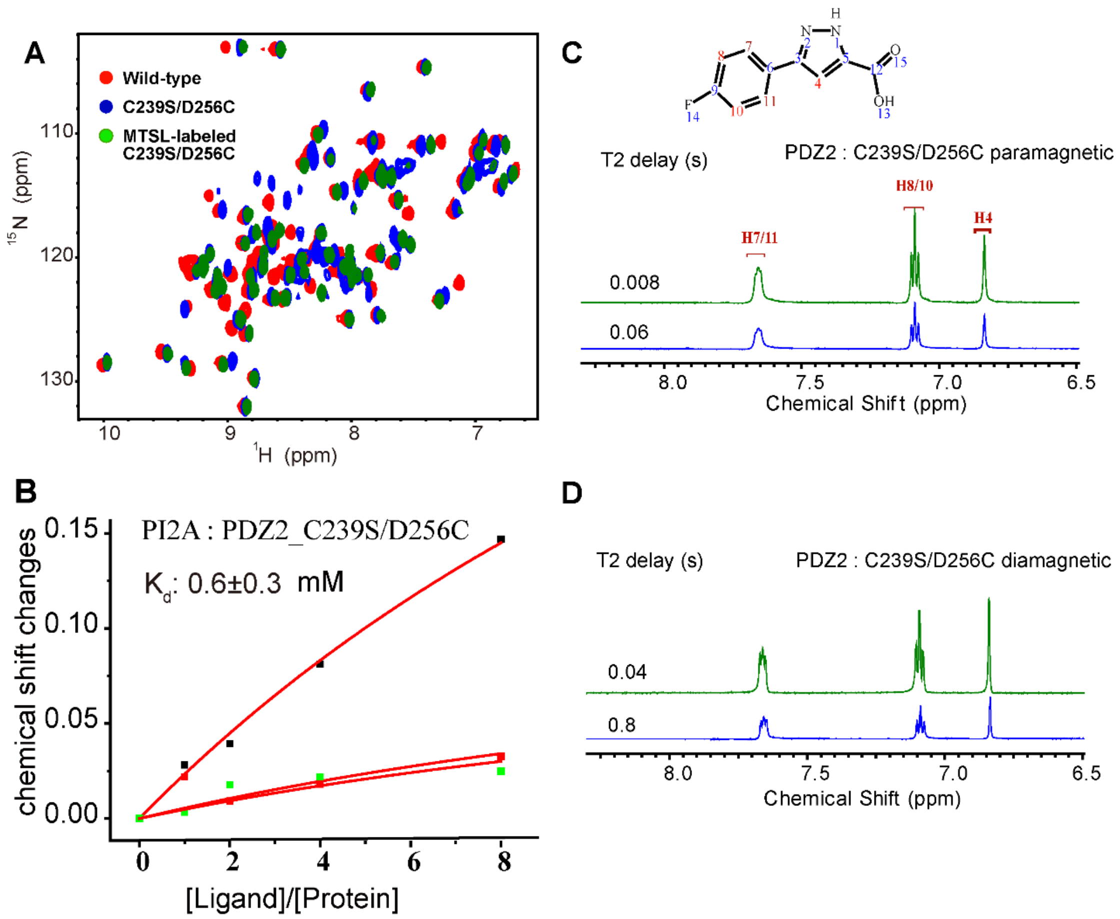

Supplementary Figure S9. Determination of PRE of PI2A transferred from MTSL-labeled MDA-9 PDZ2 domain. (A) Superimposition of ^{1}H-^{15}N HSQC spectra of the wild-type, C293S/D256C mutant, and the MTSL-labeled C293S/D256C mutant of PDZ2 domain. (B) Binding affinity between PI2A and MTSL-labeled PDZ2 domain determined by dose-dependent CSP. (C) The 1H chemical shift assignments of PI2A. Proton T2 spectrum of PI2A (0.1 mM) in the presence of MTSL-labeled MDA-9 PDZ2 domain (C) and after Vitamin C reduction (D). Annotated T2 delays.

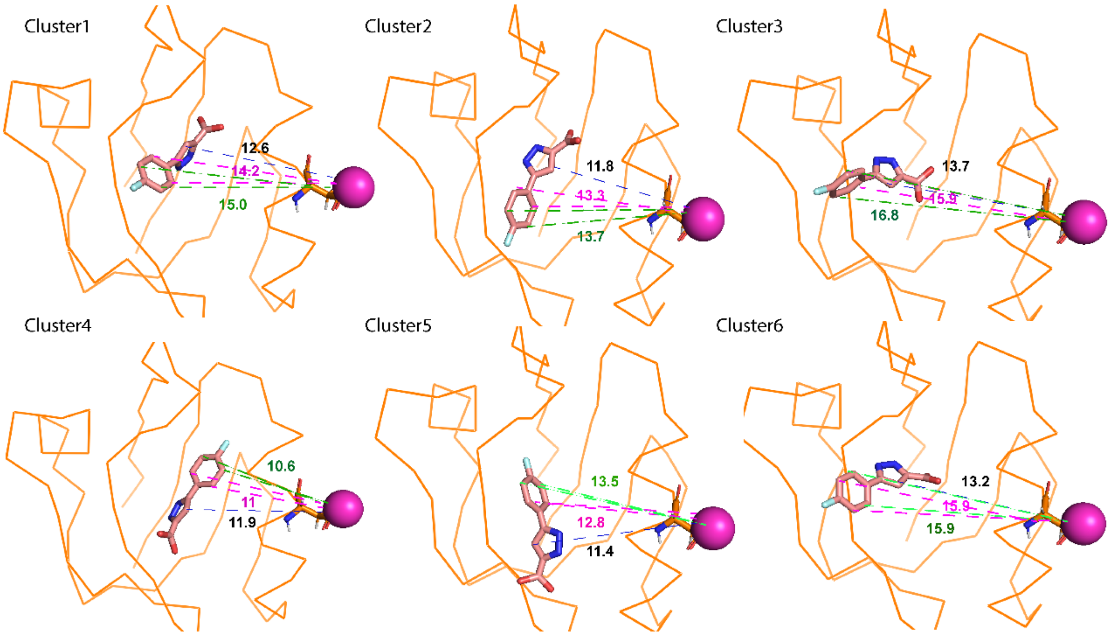

Supplementary Figure S10. The distance between the hydrogen atoms on the aromatic ring of PI2A and the paramagnetic center was measured in each docking cluster. The position of the sulfur atom (magenta sphere) of residue D256C (orange stick) of MDA-9 PDZ2 domain represents approximately the position of the nitroxide radical in the paramagnetic center. The blue dash lines represent the distances with values annotated. The green dashed lines represent the linear average of the distances from the H7 and H11 atoms to the S atom of D256C, respectively. The magenta dashed lines represent the linear average of the distances from the H8 and H10 atoms to the S atom of D256C, respectively.

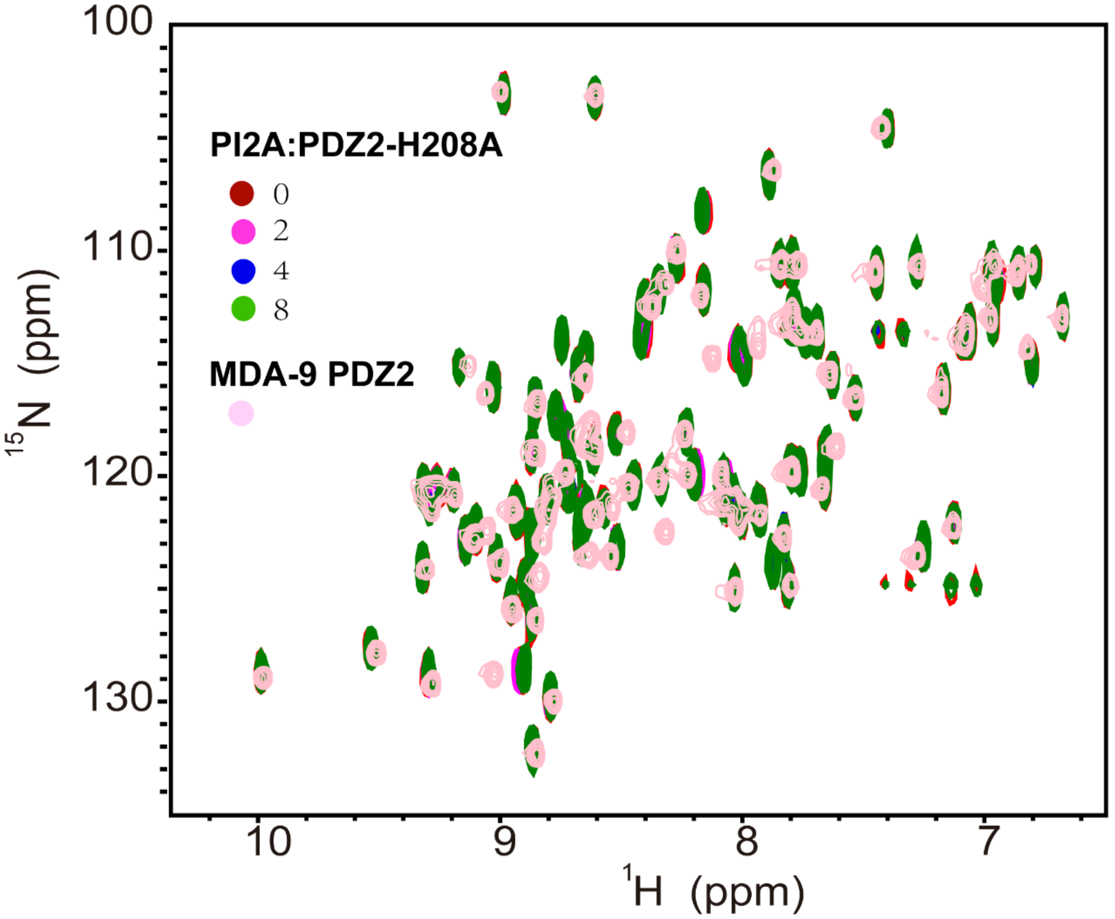

Supplementary Figure S11. Binding assays of PI2A to the H208A mutant of MDA-9 PDZ2 domain at the annotated ligand/protein molar ratios. Also, to verify the changes in the secondary structure of the proteins, the HSQC spectra of ^{15}N-labeled MDA-9 PDZ2 (pink) and the H208A mutant (red) were superimposed.

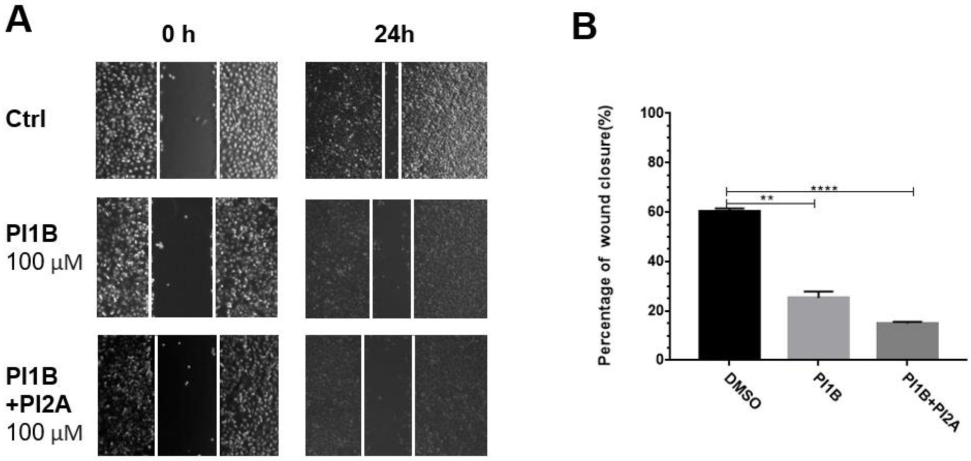

Supplementary Figure S12. Compounds PI1B and PI1B+PI2A inhibit the migration of MDA-MB-23 1 cells. (A) Wide-angle micrographs of MDA-MB-231 treated with DMSO, PI1B (100 μM) or PI1 B (100 μM) +PI2A (100 μM) for wound healing assays. (B) Wound closure relative to control (D MSO-treated cells) is indicated and the bar graph represents the mean ± SD of three independen t experiments. Statistical analysis was performed using the one-way analysis of variance (ANOV A) with a Bonferroni posttest (***P < 0.001).

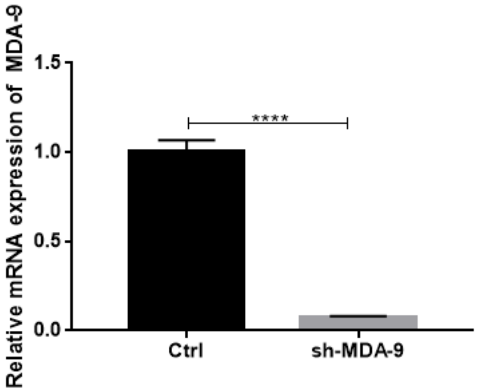

Supplementary Figure S13. knockdown of MDA-9 gene in MDA-MB-231 cells. Statistical analysis was performed using t test (****$p<0.0001$).
